# Rethinking COVID-19 and Beyond: Prevention, Remedies, and Recovery

**DOI:** 10.3389/fpubh.2022.748498

**Published:** 2022-02-23

**Authors:** Philip B. Maffetone, Paul B. Laursen

**Affiliations:** Sports Performance Research Institute NZ, Auckland University of Technology, Auckland, New Zealand

**Keywords:** overfat, obesity, health, immunity, vitamin D, glycocalyx, vaccination

## Abstract

In a relatively short timeframe, millions of deaths and illnesses associated with COVID-19 have been reported, accompanied by substantial economic losses, and overall, negatively impacting society. This experience should serve as a wakeup call to those in public health and healthcare, along with politicians and citizens: COVID-19 is considered a predictable and preventable disaster. While various reactive responses to address the pandemic were implemented, some with adverse effects, proactive measures in the years before COVID-19 were neglected. Predominately this involved the development of a preventable overfat pandemic, which played a key role in both rising rates of chronic disease, the comorbidities that increase the risk for COVID-19, along with associated inflammation and malnutrition. This increased the risk of infection in billions of people worldwide, which, in essence, primed society for high rates of COVID-19 infection. Excess body fat evolves primarily from poor nutrition, particularly the overconsumption of sugar and other refined carbohydrates, which replace the vital nutrients needed for optimal immune function. Sugar and refined carbohydrates must be considered the new tobacco, as these foods are also devoid of nutrients, and underly inflammatory chronic diseases. A balanced diet of nutrient-dense wholefood must be emphasized to combat infectious and inflammatory diseases. Implementing proactive preventive lifestyle changes must begin now, starting with simple, safe, and inexpensive dietary modifications that can quickly lead to a healthier population.

## Introduction

**Figure 1:**
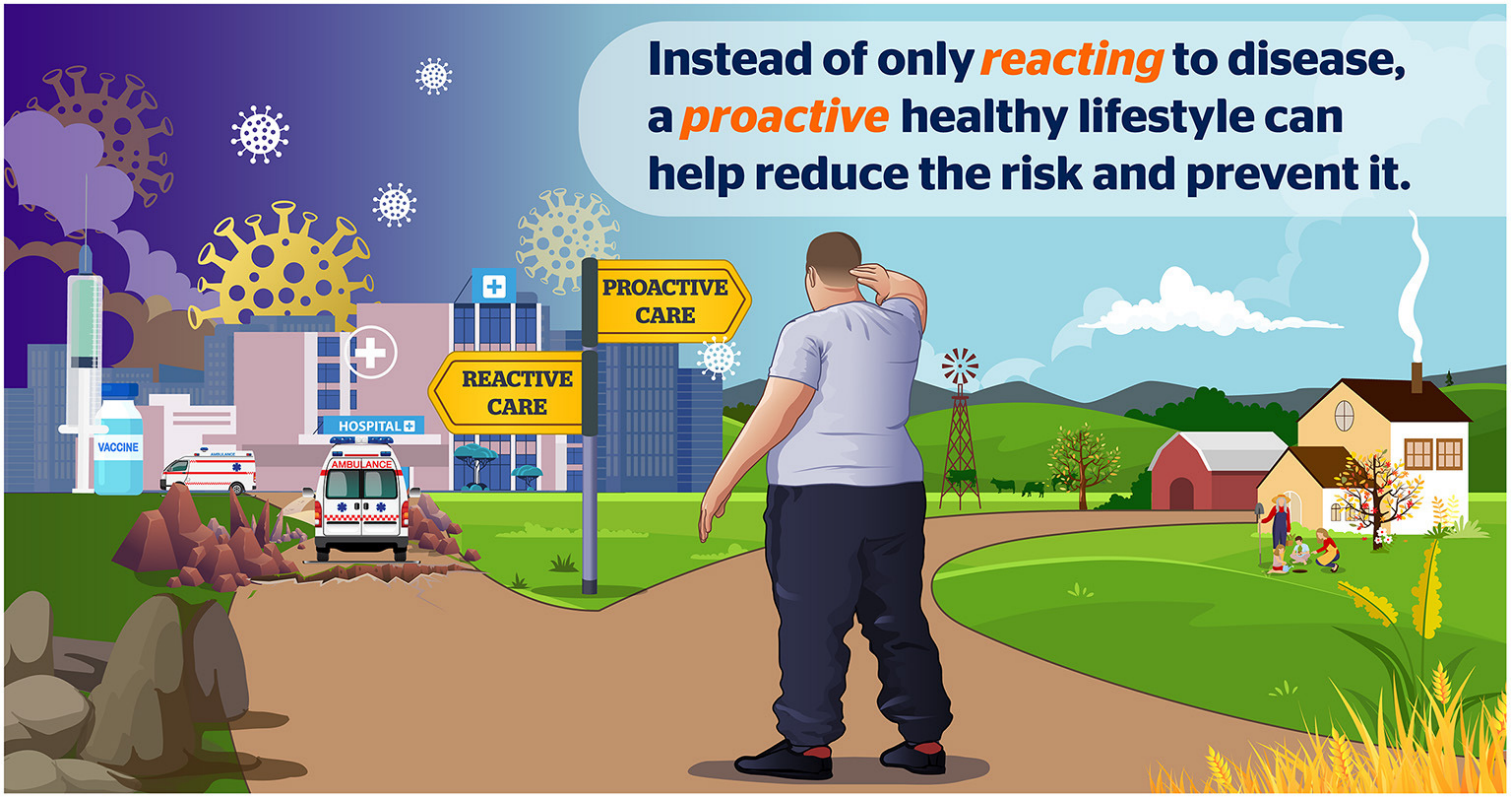
We all have a choice to make with our health. Which road will you choose?

Individuals with health risk factors, including dyslipidemia, hypertension, poor nutrition, and obesity, develop, on average, significantly more chronic disease than those with low risk. Early primary proactive prevention can help reduce or eliminate risk factors, and postpone, avert, or minimize more serious illness, disease, and premature death (1). While screening for disease can help diagnose various conditions sooner in their progression, and potentially allow for earlier treatment, this *reactive* approach is different from *proactively* preventing, reducing, and/or eliminating risk factors associated with those same diseases. Implementing a healthy lifestyle, such as improving dietary habits, can help significantly reduce disease risk, future illness, and disability (1). While both reactive and proactive approaches can be important in healthcare (Figure 1), without a logical, implementable, and proactive approach, reactive palliative care for conditions such as cardiovascular disease, diabetes, cancer, and other chronic illness must be employed, which is more expensive and leads to lower quality of life (2, 3). Unfortunately, most time and dollars in healthcare are spent on palliative care after chronic conditions are diagnosed and/or become more serious. Despite our immense knowledge of public health and lifestyle prevention, addressing the causes of preventable diseases has eluded much of today's healthcare landscape. Likewise, for the COVID-19 pandemic, which, as stated in an independent review ordered by the World Health Organization (WHO), was a “preventable disaster” (4).

While COVID-19 has been addressed as an isolated pandemic, there are in fact two related pandemics to consider. One is a serious communicable infectious disease (5), while the other is the *overfat pandemic*, an equally serious global disaster comprised of people with excess body fat (6). The overfat condition can increase susceptibility to infection due to secondary immune impairment, malnutrition, and lead to increased disease risk factors and related downstream non-communicable diseases (7, 8). In particular, the presence of excess body fat is a primary risk factor in the development of comorbidities that increase the risk of infections, including COVID-19 (7, 9, 10). Overfat has been described as the sum of obesity, overweight, plus 20–40% of non-obese, normal weight individuals who also have excess body fat (ethnicity influenced) (11), which reflects the considerable proportion of normal-weight non-obese subjects who suffer from the same metabolic conditions associated with obesity (12, 13).

**Figure 2:**
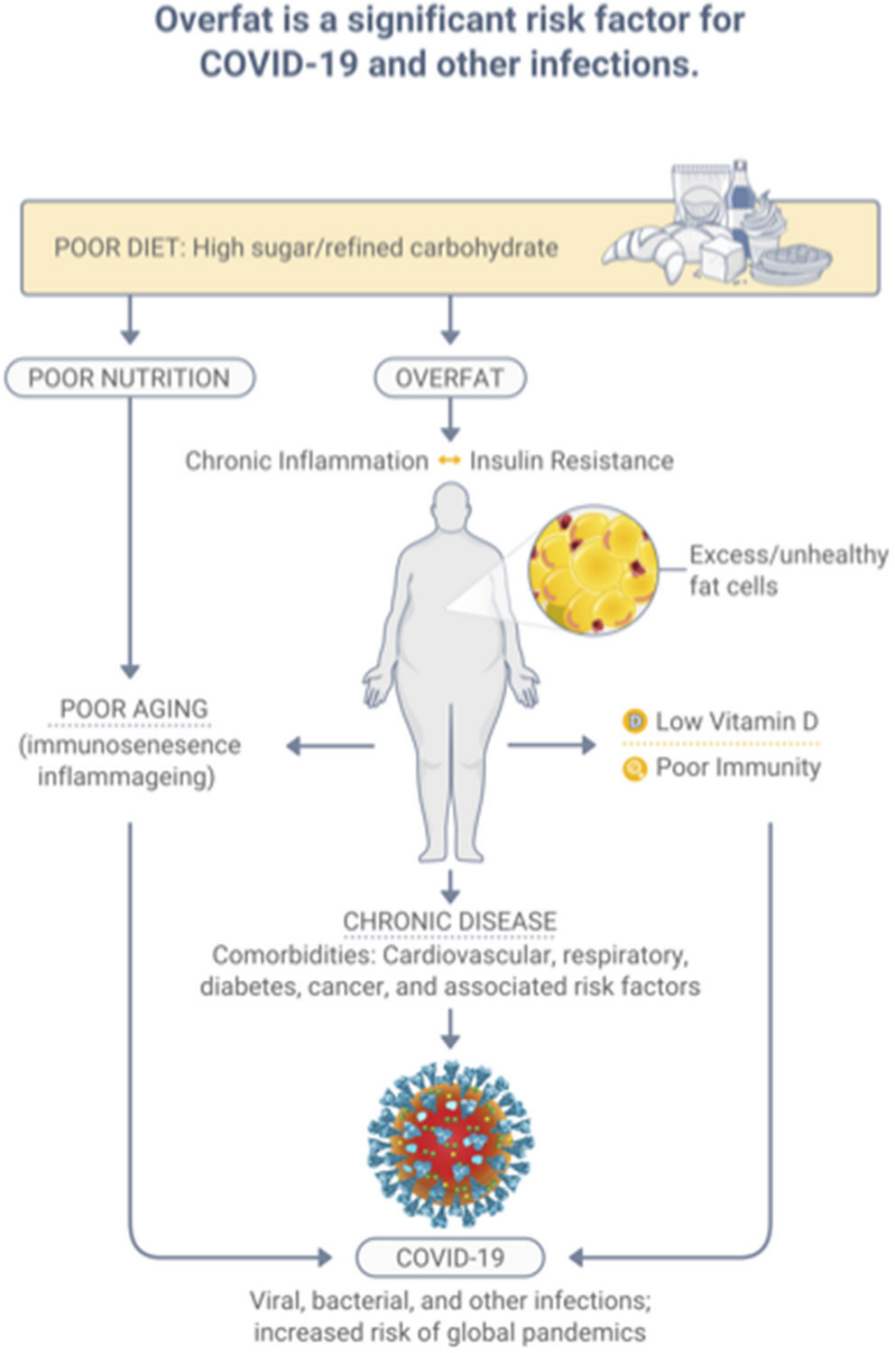
Overfat, which develops predominantly through a diet high in sugar and processed food, is a significant risk factor for infection.

The condition of overfat, which ultimately is indicative of malnutrition, adversely affects various aspects of health, including impaired innate and adaptive immune responses, promoting chronic inflammation and insulin resistance, leading to chronic diseases; comorbidities that raise the risk of COVID-19, and other infections (7). Specifically, overfat can increase COVID-19 severity and disease recovery (9, 14), and raise rates of hospitalization, admission to the ICU, the need for invasive mechanical ventilation, and the risk of mortality (14). Ryan and Caplice (15) proposed a theoretical mechanism whereby adipose tissue in obese individuals may act as a reservoir for more extensive coronavirus spread, with increased viral shedding, and cytokine amplification. A recently published systematic review and meta-analysis of 22 studies from seven countries in North America, Europe, and Asia reported that obesity was associated with an increased likelihood of presenting with more severe COVID-19 symptoms, requiring hospitalization, being admitted to an ICU, undergoing invasive mechanical ventilation, and developing acute respiratory distress syndrome compared to patients without obesity (16). Most recently, Kompaniyets et al. (17) used data from more than 800 US hospitals and found that 94.9% of 540,667 patients with COVID-19 had at least 1 underlying medical condition; predominantly essential hypertension (50.4%), disorders of lipid metabolism (49.4%), and obesity (33.0%). The strongest risk factors for death in this dataset were obesity, fear-related disorders, and diabetes (17). Figure 2 depicts the relationships between diet-induced overfat, chronic inflammation and insulin resistance, and increased chronic and infectious diseases.

As such, COVID-19 may in fact not be a pandemic. Syndemic theory recognizes that pandemics can occur in synergy with preexisting societal and health conditions, including the individual's susceptibility to disease, and would not occur, or become less serious if social and health vulnerabilities to infections were adequately reduced (18). Rather than an isolated COVID-19 pandemic, it is clear there are synergistic interactions between pre-existing biological and socioecological factors (19), the spreading of an acute infectious disease, COVID-19, fueled by the overfat pandemic and its downstream conditions including impaired immunity, malnutrition, inflammation, and comorbidities (11).

## Communicable and Non-Communicable Diseases

Global infectious diseases began diminishing drastically following improvements in public health and sanitation beginning over a century ago. However, infection rates have been increasing in frequency over the past 50 years (20). During this same period, a well-hidden preventable overfat pandemic, one that helped fuel both chronic and infectious diseases, developed. Today, over 80% of the adult world population may be overfat (11), and rates in countries such as the U.S., where the total number of confirmed COVID-19 cases and deaths is among the highest globally, 91% of the adult population was shown to be overfat (21).

In addition to chronic disease representing significant comorbidities that increase the risk of COVID-19 (and other infections), global death from chronic disease remains more than twice that of infections (22). This relationship between the prevalence of chronic and infectious diseases was shown during COVID-19's full year of 2020 in the U.S. (23). Indeed, the U.S. Centers for Disease Control and Prevention (CDC) data showed that 94% of all COVID-19 related deaths occurred in individuals who possessed an average of 4.0 pre-existing comorbidities or illnesses (i.e., obesity, existing respiratory diseases, hypertension, diabetes, cardiovascular disease, cancer, etc.) (24). As discussed below, the diet-induced overfat condition is also associated with malnutrition, which can impair immunity.

While the global prevalence of overfat exceeds 80%, the number of people worldwide with chronic diseases is also increasing, with resurging rates of infectious diseases. Therefore, addressing the overfat pandemic now may help prevent similar infectious pandemics from occurring in the future, and can significantly help reduce the global burden of disease, improve quality of life, and lower healthcare costs. Today, in the United States alone, the cumulative financial cost of the COVID-19 pandemic is so far estimated at over $16 trillion (25), while annual healthcare costs are projected to reach $6.2 trillion by 2028 (26).

## Lifestyle Behavior

As many lifestyle-related conditions significantly raise the risk of COVID-19 and other infections, the WHO suggests mandatory actions to improve patient health to reduce the risk of possible future outbreaks (27). Increased risks from tobacco use, excess body fat, and a sedentary lifestyle can promote health impairments earlier in life and have more cumulative disability at any given age than do persons with lower health risks—in other words, for the average person, reducing modifiable health risks can postpone poor health (1). However, reactive responses to COVID-19 in the form of restrictions, lockdowns, and vaccines appear to have significant adverse side-effects that can further raise the risks of infection (28). In particular, the impact of sudden sedentarism caused by home confinement and travel restrictions on the population's physical, biochemical, and mental–emotional health and fitness can lead to measurable impairment occurring in just a few days, sufficient to induce neuromuscular dysfunction, insulin resistance, lowered aerobic capacity, increased respiratory exchange ratio and fat deposition, and cause low-grade systemic inflammation (29). As an example, during COVID-19, significant weight gain suggestive of malnutrition occurred in adults (30) and children (31), potentially further increasing the risk for infection and chronic disease.

It has also been estimated that between 10 and 30% of those with a history of COVID-19 still experience debilitating symptoms months after being infected (32). Referred to as “long-haul Covid” (or “long Covid”), this may be a new public health disaster in the making (33). Importantly, a healthy lifestyle can also play a role in recovery from COVID-19 and especially for those with long Covid (34).

The COVID-19 vaccination program is a reactive public health response to the pandemic (Figure 1) with unfortunate consequences (35). Vaccines, which require a robust immune response to work properly, may be less effective in those with excess body fat due to the associated poor immunity, including those with COVID-19 (36, 37). These inadequate immune responses have been exposed as a major public health liability, and previously have not been well-recognized. In the overfat, malnourished, and immune-compromised host, the viral lifecycle can be altered, complementing an already weakened immune response, lead to severe pathogenesis and prolonged viral shedding, and can permit the emergence of virulent minor variants (36, 38, 39). In addition, shedding of the sulfate-dependent glycocalyx component of cell surfaces may also cause significant pathological consequences in COVID-19 patients (34). Addressing a weak immune system, especially in the overfat and malnourished population, is an urgent public health priority (36), and is an example of proactive healthcare (Figure 1). As du Preez et al. (34) state, “A quick fix drug and vaccine approach will not address the underlying etiological factors that made us more susceptible to COVID-19.”

Obesity is associated with severe COVID-19, and people who are obese show significantly higher neutralizing antibody titer than non-obese participants (40). As a clinical feature of overfat, insulin resistance can also impair circulating lymphocytes (41, 42). In addition to excess body fat, other modifiable risk factors can impair immunity and reduce vaccine effectiveness. While this may include aging (immunosenescence), immune decline is reduced in elderly people with improved nutritional status (43, 44). Lifestyle influences frailty as well, which is also associated with poorer outcomes from COVID-19 (45) as frailty is associated with reduced immune function and effectiveness of vaccination (46). Unhealthy aging can also increase many inflammatory mediators (inflammaging), predisposing susceptible individuals to the cytokine storm implicated in poor outcomes from COVID-19 (7). However, improved nutrition can help manage oxidative stress and chronic inflammation (47).

Not unexpectedly, other COVID-19 vaccination side-effects have also been demonstrated. Reports from phase 3 clinical trial of the mRNA-1273 vaccine against SARS-CoV-2 have provided information on both immediate and delayed injection-site reactions, both local and systemic, with most signs or symptoms resolving on average after 4–5 days (48). However, some reactions have not been consistently reported by clinicians once vaccine use was implemented, and many patients unnecessarily received antibiotic agents as treatment for these side-effects (49). The pathophysiological mechanisms underlying cases of what has been called “red arms” or “Covid arms” is still unclear, although they appear to be of an immunological/autoimmunological nature (50). Control and Prevention surveys showed reactions after the first dose, including injection site pain (68%), fatigue (31%), headache (26%), and myalgia (19%), with greater reactions after the second dose, and greater reactions in younger than in older patients (51). More serious reactions to vaccines have also been reported, including cerebral blood clots in previously healthy young adults, myocarditis (52, 53), anaphylaxis, possibly due to a nanoparticle carrier system in some vaccines (54), and sadly death (55).

Due to the rapid release of these gene-altering vaccines, the long-term consequences are unknown. The vaccine RNA has specific sequences that could fuse into pathologic prion conformations (misfolded proteins) (35, 56). Furthermore, the spike protein itself, created by the translation of the vaccine RNA, binds to the angiotensin converting enzyme 2 (ACE2) receptor, a zinc containing enzyme. This interaction has the potential to increase intracellular zinc, thereby enhancing prion conformation (56). Together, the outcome could result in serious neurological diseases including ALS, frontotemporal lobar degeneration, Alzheimer's disease, and others (56). Production of the spike protein, a toxin to the body that can desulfate the cell's glycocalyx and impair its first line immune response, has the potential to contribute to a wide range of both acute and long-term induced pathologies, such as blood disorders, neurodegenerative diseases, and autoimmune diseases (35).

The global vaccination programs have quickly evolved with significant effort and expense, with several billion COVID-19 vaccination doses now being manufactured, at costs of up to $16 per dose for Western countries (54). While the balance of safety and efficacy is a primary concern, a single finding of safety and efficacy may not be sufficient for a vaccine candidate to receive US FDA approval. Data on vaccination blocking infection transmission is now being gathered. Computer models suggest 75% efficiency (54), but considering the wide range of personal, social, and economic losses, the many unknowns such as low responders to vaccines, emergence of variant viruses, and durability of vaccine-induced protection, we must still consider the obvious: how preventive actions using simple, safe, and inexpensive preparedness through a healthier population could be a significantly better option moving forward.

## Primary Lifestyle Changes

Exercise and increased physical activity, eating a balanced diet of nutrient-dense wholefood, eliminating tobacco, moderating alcohol, and caffeine, and managing stress are among the lifestyle factors that potentially can reduce risk, and improve health and fitness. However, many individuals can also feel overwhelmed when interventions attempt to modify too many behaviors (57). Multiple recommendations can make the interventions more difficult and demanding, reduce motivation, lower implementation, and lead to poor outcomes. Instead, prioritizing lifestyle recommendations to have less or a more moderate number of behavioral changes can result in better compliance and outcomes, including enlisting those individuals with low motivation, and the ability to improve untargeted or secondary unhealthy behaviors as well (58). Improving diet and exercise are two key lifestyle factors that can significantly reduce excess body fat and improve health. While exercise recommendations alone can have inadvertent effects on food intake (59), diet alone may play a much greater role in reducing the prevalence of overfat (21, 60). Simple dietary recommendations could serve as a significant lifestyle change appropriate for an unhealthy overfat population with rising chronic disease and healthcare costs, increasing rates of infections, and a vulnerability for future infectious pandemics.

Over the past half century, traditional low-fat and low-calorie diet approaches have been a hallmark of weight-loss programs yet are unsuccessful long-term. Despite recent increases in exercise rates, the overfat pandemic has grown dramatically (21). As an alternative, research shows rapid effectiveness and healthfulness once the consumption of refined carbohydrates, including added sugars (glucose + fructose) is lowered; not only for reducing excess body fat but for lowering the risks of and treatment for cardiovascular and metabolic conditions, some cancers, and other health problems (61–64), and recently for COVID-19 (9, 65). While a low-carbohydrate high-fat diet has been used in traditional medicine for about a 100 years, for diabetics since before insulin was developed and for seizure control, the natural lifestyle of the earliest humans relied on this diet to generate large quantities of metabolic energy from the oxidation of fatty acids to develop larger brains and bodies, prevent and reduce disease risk, extend longevity, in addition to other benefits (66). However, a natural balanced wholefood diet in any ratio of complex carbohydrate, fat, and protein should be the emphasized starting point.

As sugar's influence on health is also a key driver of long-term economic growth, public expenditure, and consumer trends (67), addressing the problem may require a combination of public and private policy actions. This may include improved educational campaigns to help influence individual health behavioral changes. Referring to sugar as “the new tobacco” for its health risks (68), there is the potential of rapid and substantial health gains and cost savings with improved sugar labeling (69). Indeed, forecasts on the economic effects of sugar via computer modeling suggests that reduced sugar consumption may result in rapid and significant public health and economic benefits (70). While the political appetite for greater action may be weak, with debates around the appropriateness of government intervention (via an unpopular “sugar tax,” tax incentives for “healthy” foods, or other increased regulation), the possibility of incentivizing body fat loss for individuals has been shown to be both effective and cost saving (71).

We are suggesting that global public health guidelines also prioritize addressing current and future infectious conditions, chronic disease, and the overfat pandemic, and recovery from COVID-19, as an all-inclusive, simple, less expensive, and effective recommendation through healthy dietary habits. This can be accomplished with a consensus to approach the problem not unlike tobacco, as a product that seriously impairs population heath and the economy.

## Vitamin D, COVID-19, and Overfat

**Figure 3:**
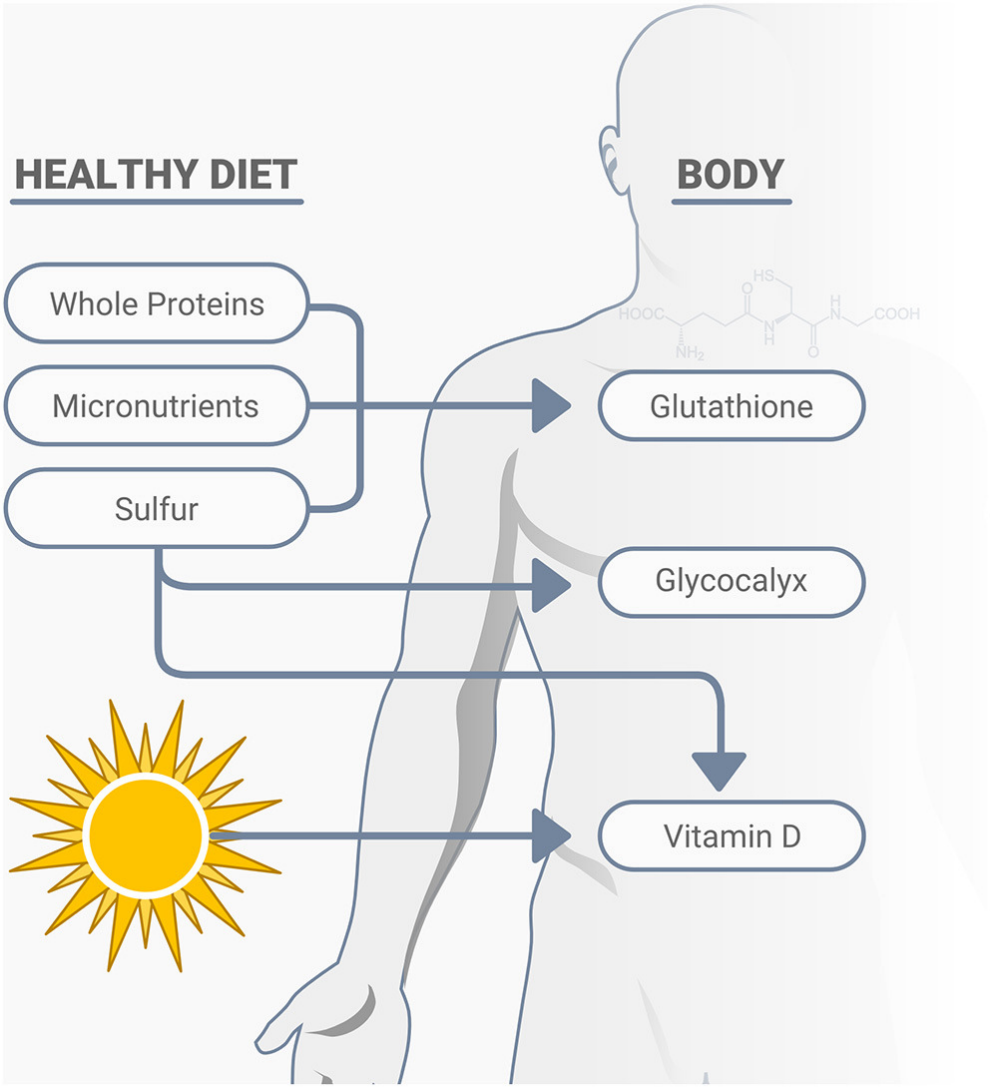
A healthy diet consisting of nutrient dense whole proteins, micronutrients and sulfur rich compounds support cellular immunity, largely through their positive effects on glutathione production, the glycocalyx, as well as the activation of vitamin D.

Vitamin D plays many roles in immune function, including cellular aspects of innate immunity, T-cell mediated immunity, B-cell mediated immunity, improved gut and skin barrier function, and others associated with antimicrobial activities (72). Low serum 25-hydroxyvitamin D (25D) concentrations, vitamin D deficiency, is another preventable global pandemic, with body fat content inversely related to serum 25D concentration and a decline in cutaneous vitamin D synthetic capacity, which is also associated with age (73–75). Sulfur deficiency is also related to low vitamin D status (34). Adequate vitamin D can affect immune modulation, reduce inflammatory cytokines, expresses anti-microbial peptides in neutrophils, monocytes, and natural killer cells, promotes innate immune activation, plays a protective role in the epithelial cells lining the respiratory tract, as well as having other important relationships with infections, including its potential impact on COVID-19 (76–79). Numerous published studies on low vitamin D status and COVID-19 show the potential for significant benefits of this overlooked and inexpensive assessment and treatment option (80–86). While vitamin D deficiency can impair the response to seasonal influenza vaccinations (87), it is not yet known whether this includes the COVID-19 vaccines. In addition to vitamin D, other nutrients may also have important relationships with COVID-19. The early discovery that SARS-CoV-2 engages the ACE2 for entry into the cell for infection has prompted increased research efforts to elucidate the biochemical determinants of CoV-ACE2 interactions (88). Various natural compounds found in a healthy diet may impact positively on these interactions and serve as adjunctive treatments, including the micronutrient zinc (88), and vitamins C and E (55), thiamine, along with quercetin and other phytonutrients (89, 90). In addition to ACE2, cell surfaces also depend on sulfur, as the degree of sulfation of heparan sulfate may play a primary role in the risk of infection and specifically SARS-CoV-2 entrance into human cells, along with its influence on immunity, inflammation, and oxidative stress (34, 91, 92). du Preez et al. (34) state that, “Undersulfation (less than the normal degree of sulfation) or aberrant sulfation of HS [the degree of sulfation of heparan sulfate] may not only increase susceptibility to viral infection but may also adversely affect the individual's physiological response to the infection.” A variety of naturally occurring nutrients impact immunity and resistance of infections, oxidative stress, and excess inflammation, including sulfur and its related amino acid cysteine, various micronutrients, vitamin D, and others (93). A sulfur-rich natural diet that includes cruciferous vegetables and whole proteins, including grass-fed animal products, helps increase glutathione production, the body's most powerful antioxidant (Figure 3). A notable concern is the great push for “synthetic or cultured/pant-based” meat, which is low in sulfur-amino acids, and will not replace the health and immunological properties of real grass-fed animal meat.

Another benefit of reducing sugar and other refined carbohydrates is that junk food can replace many other nutrient-dense foods that would typically provide a wide range of natural macro-, micro, and phytonutrients, and otherwise impair one's nutritional status.

## Discussion

Early reactive responses to COVID-19 may have been necessary emergency procedures. However, one could argue that preventive measures with a focus on improving health could have also potentially accomplished the same, only better, as the strategy is cheaper, and could help prevent or reduce the severity of a pandemic. Whether we waited too long to respond to the COVID-19 outbreak, or not, we still must consider what proactive, preventable approaches could have better influenced such a familiar and predictable disaster, as medical and public health communities have long warned of the potential for such a pandemic (94). COVID-19 has revealed a world that was unprepared, overconfident, and inept in pandemic control, especially in the U.S., with its trillion-plus dollar annual healthcare budget, extensive infectious disease monitoring and research, and academic and pharmaceutical capacity. One way to address this problem is to quickly and significantly reduce the primary overfat pandemic by promoting and making available nutrient dense foods for society.

**Figure 4:**
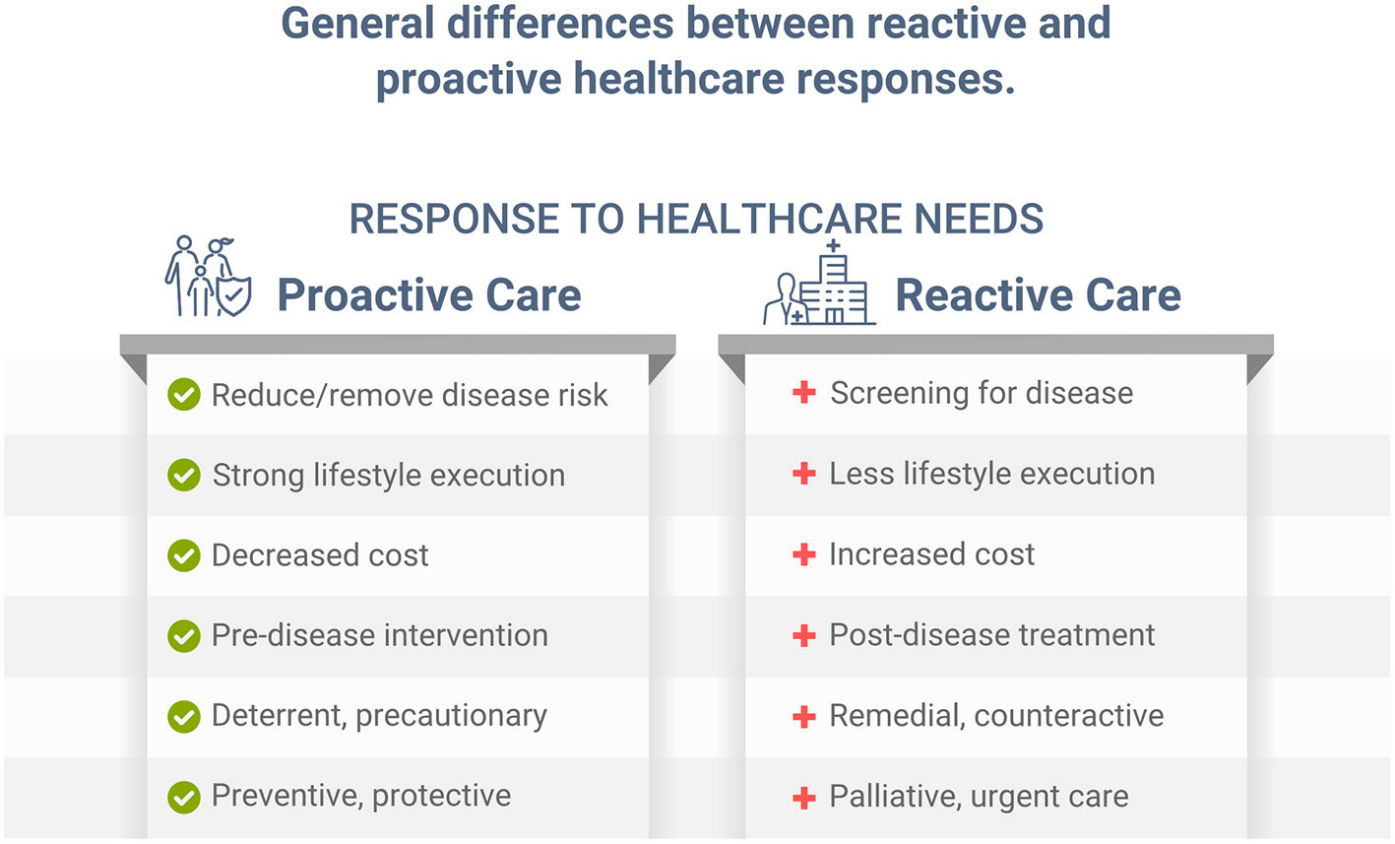
The general difference between reactive and proactive healthcare responses.

The immense overfat global population has a growing impact on both non-communicable and communicable diseases. It is obvious that we must address the long-standing overfat pandemic and its spawning comorbidities that helped promote COVID-19, which may also increase the risk of the next global infectious pandemic. Combining the biological, social, psychological, and other factors to accomplish this task would be like addressing the health effects of tobacco. However, tackling the tobacco problem was a very long slow process due to political, financial, and social issues; and it remains a serious global health problem today. Instead, a rapid, synergistic approach using all our knowledge of public health, clinical, and scientific resources to recover from the COVID-19 pandemic and its fallout, reduce the overfat pandemic and its downstream comorbidities, and prevent future pandemics, can be far more successful than waiting for disease to occur then trying to react to it (Figure 4).

This immediate public health focus is an urgent and necessary step following our lessons from COVID-19. Rather than keep repeating the many general lifestyle recommendations most generations have been exposed to during the past half century, when preventable conditions such as the overfat pandemic exploded, and infectious and chronic disease rates rose, it may be best to consider a single and simple dietary approach of markedly reducing refined carbohydrates, including sugar, as a primary step to help quickly change population health, which can help reduce excess body fat and downstream conditions. This would require a global effort, despite the economic impact on certain industries that produce unhealthy food products. While this may initially appear to be a radical approach, too little has been done in the past, and we cannot afford another COVID-19 pandemic, especially considering that it could be much worse than the current one.

There will be no victory over COVID-19: it has negatively impacted the mental and physical health of society, and we have incurred significant economic loss. The experience should serve as a wakeup call to all those in public health—and the rest of the world, including individuals, who are most responsible for their own lifestyle choices, and governments who influence our health in many ways, especially by allowing unhealthy foods to flourish. With a rising global population, one that is also rapidly aging, and an increasing need for healthcare products and services for conditions mostly deemed preventable, true prevention can be rapid, highly effective, and an inexpensive alternative whose time has finally come.

## Data Availability Statement

The original contributions presented in the study are included in the article/supplementary material, further inquiries can be directed to the corresponding author/s.

## Author Contributions

PL and PM conceived the idea for the manuscript through discussion and observation of the response to the COVID-19 pandemic in light of the overfat pandemic. Both authors contributed to the article and approved the submitted version.

## Conflict of Interest

PM is proprietor of https://philmaffetone.com/, while PL is proprietor of https://hiitscience.com/. Both are for profit companies that serve to promote health and wellbeing for its customers.

## Publisher's Note

All claims expressed in this article are solely those of the authors and do not necessarily represent those of their affiliated organizations, or those of the publisher, the editors and the reviewers. Any product that may be evaluated in this article, or claim that may be made by its manufacturer, is not guaranteed or endorsed by the publisher.
